# Trends in Micronutrient Interventions, Anemia, and Iron Deficiency among Women and Children in Guatemala, 2009–2019

**DOI:** 10.1016/j.cdnut.2023.101970

**Published:** 2023-07-20

**Authors:** Lucas Gosdin, O Yaw Addo, Mireya Palmieri, Karla Mesarina, Dora Inés Mazariegos, Carolina Martínez, Maria Claudia Santizo, Lizet Guzmán, Yma Alfaro, Rafael Flores-Ayala, Maria Elena D. Jefferds

**Affiliations:** 1 International Micronutrient Malnutrition Prevention and Control (IMMPaCt) Program, Nutrition Branch, Division of Nutrition, Physical Activity, and Obesity, National Center for Chronic Disease Prevention and Health Promotion, Centers for Disease Control and Prevention (CDC), Atlanta, GA, United States; 2 Nutrition and Micronutrients Unit, Institute of Nutrition of Central America and Panama (INCAP), Guatemala City, Guatemala; 3 Nutrition, United Nations Children’s Fund (UNICEF), Guatemala City, Guatemala; 4 Secretariat of Food and Nutrition Security, Guatemala City, Guatemala; 5 Office of Health and Education, United States Agency for International Development (USAID), Guatemala City, Guatemala

**Keywords:** Guatemala, food fortification, iron deficiency, anemia, wheat flour fortification, sugar fortification, maize flour

## Abstract

**Background:**

Food fortification and micronutrient supplementation are public health strategies to improve micronutrient status in Guatemala; their population effectiveness has not been evaluated in recent years.

**Objective:**

We evaluated trends in food fortification, micronutrient supplementation, anemia, and iron deficiency among nonpregnant women aged 15–49 y [women of reproductive age (WRA)] and children 6–59 aged mo [preschool age children (PSC)].

**Method:**

Nationally representative serial cross-sectional surveys were used to assess changes in hemoglobin, anemia, ferritin, iron deficiency, iron deficiency anemia, and self-reported consumption of fortifiable foods and micronutrient supplements during 2008/2009, 2013, 2015, 2016, 2017/2018, and 2018/2019. Predictors of hemoglobin and ferritin were assessed using generalized linear mixed models adjusted for survey year as random effects, and the consumption of fortifiable foods, supplements, and other potential confounders were fixed effects.

**Results:**

Multiple micronutrient powder consumption among PSC during the previous 3 mo was 53.3% (95% CI: 49.4, 57.2) in 2013 and 33.6% (28.8, 38.4) in 2018/2019. Anemia among PSC was 11.3% (8.0, 14.5) in 2008/2009 and 6.1% (3.6, 8.6) in 2018/2019. Anemia among WRA was 10.7% (7.2, 14.2) in 2008/2009 and 3.9% (2.7, 5.2) in 2018/2019. Iron deficiency among PSC was 15.5% (12.1, 19.0) in 2008/2009 and 10.9% (7.4, 14.5) in 2016 (lowest), but 17.1 (13.3, 21.0) in 2017/2018 (highest). Iron deficiency among WRA was 14.9% (11.6, 18.2) in 2008/2009, 13.8% (11.8, 15.8) in 2013 (lowest), and 18.9% (16.3, 21.6) in 2017/2018 (highest). Wheat flour/bread consumption was positively associated with hemoglobin among PSC, and sugar consumption was positively associated with hemoglobin among WRA. The reported consumption of fortifiable foods was not associated with ferritin among PSC or WRA.

**Conclusions:**

Guatemala has implemented multiple food fortification strategies, and anemia has declined. Increases in iron deficiency in 2017–2019 warrant further attention. Secular trends toward poverty alleviation, education, and development might be responsible for changes not explained by the micronutrient interventions evaluated.

## Introduction

Affecting an estimated 1.74 billion people worldwide, anemia is a major cause of morbidity globally resulting in an estimated 58.6 million years lived with disability in 2019 [1]. The condition results from multiple etiologies, including both micronutrient deficiencies and nonnutritional causes, such as illness or blood loss [2]. Iron deficiency is a common cause of anemia in most populations, although causes vary by population [3]. Vitamins A, B12, and folate are other micronutrient causes, among others [4,5]. Anemia is associated with poor health and economic outcomes, and related micronutrient deficiencies have additional negative consequences, which makes addressing them a public health priority [4,6].

Guatemala currently mandates fortification of salt and 4 staple foods: salt, sugar, wheat flour, and nixtamalized maize flour (Supplemental Table 1), the last one was added to Guatemala’s fortification portfolio in 2016 by the government mandate, meaning that 2 staple foods (wheat and maize flour) have been mandatorily fortified with iron [7]. A popular brand of sugar in Guatemala voluntarily began fortifying with iron (ferric amino acid chelate) in late 2008 [8]. Specialty cereal products, including some provided by the government, are also voluntarily fortified with iron [9]. Guatemala’s efforts to strengthen enforcement of its fortification mandates are well documented and include the establishment of the National Commission for the Fortification, Enrichment, and/or Equalization of Foods, which coordinates and supervises food fortification programs [9].

Because, in general, young children do not consume industrially fortified foods in sufficient quantity, micronutrient supplementation is also used for prevention in this population. In Guatemala, high-dose vitamin A supplements were administered biannually to preschool-aged children 6–59 mo from the early 2000s to 2014, after which it was determined that there was sufficient evidence of adequate year-round vitamin A intake and low deficiency to limit biannual supplementation to only those aged 6–24 mo [10], and it was further limited to those aged 6–11 mo in 2018 [11]. Since 2010, multiple micronutrient powders (MNPs) containing iron, folic acid, zinc, copper, selenium, iodine, and vitamins A, B1, B2, B3, B6, B12, C, D, and E have been distributed for home fortification of foods given to children aged 6–59 mo. Unlike mass fortification, home fortification requires more intensive interventions at the consumer level aimed at behavior change and demand generation [12].

National nutrition surveys in Guatemala before 2013 showed a gradual decline in anemia, although these surveys occurred with long intervals of 5–10 y between them and only one in 2008/2009 evaluated micronutrient deficiencies. These national surveys did not evaluate industrially produced fortifiable food consumption and micronutrient content of fortifiable foods. Changes in indicators and methodologies also create additional issues with comparing these data over time. To resolve these issues, the Integrated Reproductive Health and Nutrition Surveillance System (Sistema de Vigilancia Epidemiológica de Salud y Nutrición [SIVESNU]) was created [13].

Using the national micronutrient survey from 2008/2009 (Encuesta Nacional de Micronutrientes, ENMICRON) as an anchor and SIVESNU serial cross-sectional survey data from 5 cycles between 2013 and 2019, we conducted a secondary data analysis of trends in anemia, micronutrient status, micronutrient supplementation, and fortification. Our study was guided by a program impact pathway for population-based fortification programs [14]. The impact pathway outlines key indicators for monitoring and evaluating food fortification programs. Indicators include the availability, quality, coverage within households, consumption by individuals, and micronutrient contribution of fortified foods [14]. We hypothesized that fortification programs in Guatemala resulted in staple foods with higher iron, vitamin A, and other micronutrient contents, and the consumption of fortifiable foods and micronutrient supplements was associated with higher levels of hemoglobin and serum ferritin concentration during the 10-y period.

## Methods

### Setting and sampling frame

Survey protocols were approved by either the Institute of Nutrition of Central America and Panama Institutional Ethics Committee and/or the Ethical Review Committee of the Ministry of Health of the Republic of Guatemala. All individuals gave informed consent and caregivers gave informed consent for children aged 6–59 mo before participation in all surveys. National surveys were conducted in Guatemala in 2008/2009 (ENMICRON), 2013, 2015, 2016, 2017/2018, and 2018/2019 (SIVESNU). All nonpregnant women of reproductive age (WRA), classified as females aged 15–49 y, and preschool age children (PSC), classified as children aged 0–59 mo, were eligible for inclusion (aged 6–59 mo for biomarker collection). Detailed information on the sampling methodology for each survey is reported elsewhere [13]. Briefly, these nationally representative household surveys used multistage cluster sampling designs and selected clusters with probability proportional to their number of households. For ENMICRON 2008/2009, the Guatemalan National Statistics Institute 2002 sampling frame was first stratified by regions and rural–urban and selected 734 clusters for the National Maternal and Child Health Survey (ENSMI) 2008. ENMICRON 2008/2009 drew a subsample of 246 clusters from the 734 ENSMI clusters. For each year of SIVESNU, 100 clusters were selected from the Guatemalan National Statistics Institute 2002 sampling frame. Thirty households were randomly selected from each cluster. One WRA and one PSC were randomly selected from each household. Pregnant WRA were excluded from this analysis.

### Questionnaires

An adult household member reported information on household demographics and expenditures. Household food expenditures focused on the quantity and types of sugar, bread, and maize flour. Separate questionnaires collected information from WRA and primary caregivers of PSC on demographics and consumption of fortifiable foods and micronutrient supplements. The fortifiable food consumption was assessed in terms of type and frequency of consumption by the individual in the home within the previous 24 h and 7 d. The individual consumption of fortifiable foods was expressed as the number of days the food was consumed over the previous 7 d categorized as 0, 1–2, and 3–7 d. Fortifiable foods were categorized into the following 4 groups: *1*) sugar or sugar-sweetened foods or beverages, *2*) bread or wheat flour, *3*) fortified cereals including Incaparina and VitaCereal, and *4*) maize flour (not including tortillas made outside the home). Household poverty was assessed by the Global Multidimensional Poverty Index (MPI) and categorized into 4 levels of intensity: *1*) no poverty, *2*) vulnerable to poverty, *3*) poverty, and *4*) severe poverty [15]. The Global MPI has been used in Guatemala and other low- and middle-income countries as an objective and comparable measure of poverty. Current use of reversible contraceptives among WRA, dichotomized as current or noncurrent users, included the use of intrauterine device, contraceptive injection or implant, oral contraceptive, vaginal contraceptive method, or hormonal contraceptive patch in the previous 30 d.

### Anthropometry

Standard anthropometric techniques were followed in the assessment of weight and height/length [13,16]. BMI was calculated for WRA, and international cut-offs for underweight (BMI <18.5 kg/m^2^), overweight (BMI 25 to <30 kg/m^2^), and obesity (BMI ≥30 kg/m^2^) were applied [17]. BMI-for-age *z*-scores (BMIZ), height-for-age *z*-scores (HAZ), and length-for-age *z*-scores (LAZ) were calculated using the WHO growth standards for PSC [18].

### Biochemical assessments

A full accounting of sample collection, processing, and assays can be found in the survey reports [[19], [20], [21], [22], [23], [24]]. Nonfasting blood specimens were collected by highly trained staff. Hemoglobin concentration (Hb) was assessed by HemoCue 301 using pooled capillary (years 2013 and 2015) and venous (years 2008/2009, 2016, 2017/2018, and 2018/2019) blood samples. Anemia was defined as Hb <11 g/dL for PSC, adjusted for altitude, and Hb of <12 g/dL for WRA, adjusted for altitude and smoking [25]. Serum ferritin concentration was assessed by automated chemiluminescent immunometric assay (Immulite 1000 MAGLUMI) in 2008/2009 and by ELISA in all other years [26]. In 2008/2009, α-1-acid glycoprotein (AGP) was assessed by the turbidimetric method. For the 5 SIVESNU cycles, AGP, ferritin, sTfR, CRP, and retinol-binding protein (RBP) were assessed in combination by a sandwich ELISA [26]. CRP, sTfR, and RBP were only available in the 5 SIVESNU cycles. Serum retinol was assessed by HPLC in ENMICRON 2008/2009 [27]. Serum zinc was assessed by atomic emission spectrometry [28] among PSC in 2008/2009 and 2013 (random subsample), and 2016 (random subsample) and among WRA in 2013 (random subsample) and 2016 (random subsample). Plasma folate was measured in 2008/2009 using a microbiologic assay [29]. Serum vitamin B12 was assessed by chemiluminescence in 2008/2009 and 2016 [30].

Ferritin was inflammation adjusted for CRP and AGP using regression correction, except for ENMICRON 2008/2009, where AGP alone was used as CRP was unavailable [31]. Iron deficiency was defined as inflammation-adjusted ferritin <12 μg/L for PSC and ferritin <15μg/L for WRA [32]. Iron deficiency anemia was defined as concurrent anemia and iron deficiency. The cut-point for elevated sTfR indicating iron deficiency erythropoiesis was an inflammation-adjusted sTfR >8.3 mg/L [33]. Vitamin A deficiency was defined as a serum retinol <0.7 μmol/L or an RBP <0.7 μmol/L as retinol equivalents (inflammation adjusted among PSC only) [27,34]. Zinc deficiency was defined as serum zinc <65 μg/dL for PSC (inflammation adjusted) and <66 μg/dL for WRA; unknown collection time prevented applying time-specific cut-points [28,35]. Folate deficiency was defined as serum folate <3 ng/mL [29]. Low vitamin B12 was defined as serum B12 <200 pg/mL [30].

### Food samples

Samples of bread and sugar were collected in each of the SIVESNU cycles. Complete details of the sample collection, processing, and analysis of food samples have been published previously [[19], [20], [21], [22], [23]]. A sample of bread was collected from the local bakery or store where bread was reported to be most often purchased by households within each cluster. Bread was not collected from households, whereas standard (white) sugar was collected from a random subsample of households. Vitamin A content was assessed by the quantitative spectrophotometric method. Iron was measured in sugar samples by acid microwave digestion, followed by atomic emission spectrometry. Iron in bread and retinol in sugar were compared with regulatory minimums: 33 and 5 mg/kg, respectively [36,37]. Iron in sugar was compared with the 6 mg/kg that producers reported as the minimum level of voluntary fortification reported by the manufacturer [8].

### Statistical analyses

Descriptive statistics (means and percentages) were calculated for characteristics of WRA and PSC within each survey year. The household-level coverage of 6 fortifiable foods, the presence in the household, quantity purchased, and the country of origin were described using medians with interquartile range or percentage. Across the years, differences in the population prevalence were examined using survey design-adjusted Rao–Scott chi-square tests, and differences and trends in mean continuous variables were tested using design-adjusted linear regression. Kruskal–Wallis tests were used to examine nutrient content of fortifiable foods from bakeries and households in terms of iron in bread and sugar, and retinol in sugar, which were compared across the survey years. Crude prevalence estimates across survey years of anemia and anemia-related micronutrient deficiencies were visualized with plots.

#### Trend analyses and model covariates

Overall trends in anemia, Hb, iron deficiency, (log-transformed) ferritin, and iron deficiency anemia were assessed separately by survey linear regression (identity link) with survey year as the sole fixed effect [38]. We applied complex survey linear regression models to study trends in Hb and ferritin, and their associations with participant-, household-, and population-level covariates over the survey years. In these multivariate generalized linear mixed effects models (GLMMs), adjusted secular trends were captured as survey year with a random intercept. Participant-level characteristics were modeled as fixed effects of the studied population during each year. Model covariates (confounders and effect modifiers) were selected based on prior literature and included age, parity (WRA only), sex (PSC only), ethnicity, rurality, remittances (WRA only), education (WRA only), BMI, LAZ/HAZ (PSC only), household poverty, RBP, current use of reversible contraceptives (WRA only), frequency of fortifiable food consumption, and micronutrient supplementation (PSC only). Because of the nearly universal consumption of sugar in the surveys, only standard sugar (azúcar estándar), fortified with vitamin A and potentially fortified with iron, was used as a covariate in GLMMs.

Associations between the covariates and nutritional indicators are presented in g/dL for Hb and percentage change for ferritin. Average survey year (random slopes) effects were calculated as best linear unbiased predictors (BLUPs). The use of BLUP estimates enabled the adjustment for the intersurvey and intrasurvey variations in biomarker distributions and are interpreted as the difference in population mean Hb or ferritin from the grand marginal mean (intercept) of all participants over all survey years [39]. Bootstrapped estimates of 500 iterations were used to generate 95% CIs around the BLUPs.

Given potential differences in fortification levels across the years (effect modification), separate GLMMs containing a statistical interaction term of fortifiable food consumption and survey year were used to assess differential effects.

Sampling weights were applied where indicated. Statistical significance was set at *P* value of < 0.05 with 95% CI as needed. All analyses were conducted in SAS 9.4 (SAS Institute).

## Results

### Basic characteristics

The 6 nationally representative surveys have a combined sample size of 4133 PSC and 9008 WRA. These samples represent ∼900,000 PSC and 4 million WRA during each year of the study period [40]. Table 1 compares selected demographic and nutrition-related characteristics of PSC across the study period. The distribution of age, sex, ethnicity, and rurality were consistent across time (*P* = 0.08, 0.98, 0.69, and 0.08, respectively). Poverty decreased over the studied period (*P* < 0.001). For example, the proportion experiencing poverty and severe poverty were 27.6% and 22.1%, respectively, in 2008/2009 and 22.9% and 8.8%, respectively, in 2018/2019. Mean BMIZ declined over time from 0.47 in 2008/2009 to 0.39 in 2018/2019 (*P* = 0.02), whereas mean HAZ/LAZ did not vary significantly remaining between −1.85 and −2.01 (*P* = 0.38). The decline in high-dose vitamin A supplementation was precipitous, from a high of 68.1% in 2013 to a low of 7.6% in 2018/2019, corresponding to the 2014 and 2018 changes to stop high-dose vitamin A supplementation for children aged ≥12 mo. Reported receipt of MNPs during the previous 3 mo ranged from a high of 53.3% in 2013 to a low of 11.3% in 2016. Supplementation with iron and folic acid was low across all surveys.

**TABLE 1:** Characteristics of preschool age children in nationally representative surveys in Guatemala

	2008/2009/2010^1^ (*n* = 871)	2013^2^ (*n* = 878)	2015^2^ (*n* = 664)	2016^2^ (*n* = 577)	2017/2018^2^ (*n* = 546)	2018/2019^2^ (*n* = 597)	*P* value^3^
Age (%)							0.08
6–24 mo	38.0	33.4	31.5	34.0	32.8	31.3	
25–59 mo	62.0	66.6	68.5	66.0	67.2	68.7	
Female (%)	49.9	49.9	48.0	51.5	49.8	49.5	0.98
Indigenous (%)	38.8	44.9	35.1	36.4	39.8	43.9	0.69
Area (%)							0.08
Urban	38.8	24.8	41.0	30.0	34.9	33.3	
Rural	61.2	75.2	59.0	70.0	65.1	66.7	
Household receives remittances (%)	—	8.0	12.6	12.9	13.0	15.3	0.01
Multidimensional poverty index (%)^4^							<0.001
No poverty	—	31.5	36.7	34.0	44.2	47.6	
Vulnerable to poverty	—	18.9	23.4	23.1	17.6	20.6	
Poverty	—	27.6	28.9	28.1	25.3	22.9	
Severe poverty	—	22.1	11.1	14.8	12.8	8.8	
BMI-for-age *z*-score, mean	0.47	0.50	0.30	0.33	0.37	0.39	0.02
Length-for-age/height-for-age *z*-score, mean	−1.87	−1.95	−1.85	−2.01	−1.85	−1.89	0.38
High-dose vitamin A supplement in previous 6 mo (%)	31.8	68.6	29.9	19.5	16.6	6.9	<0.001
Iron supplement in previous 3 mo (%)	—	12.5	3.6	2.2	2.1	3.1	<0.001
Folic acid supplement in previous 3 mo (%)	—	10.9	4.2	0.0	1.6	2.7	—
Multiple micronutrient powders in previous 3 mo (%)	—	53.3	31.1	11.3	23.3	33.6	<0.001

Weighted estimates.

1 Encuesta Nacional de Micronutrientes.

2 Sistema de Vigilancia Epidemiológica de Salud y Nutrición.

3 Rao–Scott chi-square test (categorical variables) or ANOVA with Taylor series variance (continuous variables) for differences in distribution within at least one survey cycle.

4 The Multidimensional Poverty Index is an international measure capturing acute deprivations in health, education, and living standards [48]. It is a household-level indicator, though differences between estimates for PSC and WRA exist because not all households with WRA have PSC.

Table 2 compares selected demographic and nutrition-related characteristics among WRA. The distribution of age and the proportion of indigenous women were unchanged across the surveys (*P* = 0.80 and 0.85, respectively). Parity decreased, whereas the number of years of schooling increased (*P* < 0.001). Poverty decreased (*P* < 0.001), and the distribution of BMI shifted toward obesity (*P* = 0.001). For example, 18.6% had obesity in 2008/2009 and 25.9% had obesity in 2018/2019.

**TABLE 2:** Characteristics of nonpregnant women of reproductive age in nationally representative surveys in Guatemala

	2008/2009^1^ (*n* = 1323)	2013^2^ (*n* = 1672)	2015^2^ (*n* = 1512)	2016^2^ (*n* = 1493)	2017/2018^2^ (*n* = 1499)	2018/2019^2^ (*n* = 1509)	*P* value^3^
Age (%)							0.80
15–19 y	21.6	25.1	20.1	22.7	21.4	20.2	
20–29 y	32.9	33.3	33.6	33.1	31.8	32.3	
30–39 y	26.4	25.1	26.6	25.6	26.9	25.7	
40–49 y	19.1	16.5	19.7	18.6	19.9	21.8	
Parity							<0.001
1–2 births	36.5	45.0	43.3	46.1	45.8	46.5	
3–4 births	25.9	31.1	35.6	30.7	32.7	33.6	
5–6 births	17.0	13.4	11.9	14.1	12.7	12.8	
7+ births	20.6	10.5	9.2	9.1	8.8	7.1	
Indigenous (%)	37.2	40.3	34.2	32.8	35.3	35.8	0.85
Area (%)							0.03
Urban	48.3	33.8	48.1	36.8	39.0	42.2	
Rural	51.7	66.2	51.9	63.2	61.0	57.8	
Years of schooling (%)							<0.001
0	21.9	16.2	15.7	13.1	14.5	12.2	
1–3	23.1	19.8	15.6	17.7	17.0	16.2	
4–6	23.1	31.1	30.0	27.3	26.8	29.2	
7–9	13.2	14.1	15.7	18.9	16.5	17.6	
10–12	13.5	14.9	18.9	17.0	19.5	20.0	
13+	5.2	3.9	4.1	6.0	5.7	4.9	
Household receives remittances (%)	14.4	9.5	12.4	11.1	12.0	13.9	0.08
Multidimensional poverty index (%)^4^							<0.001
No poverty	—	53.4	56.0	52.1	58.1	61.3	
Vulnerable to poverty	—	17.2	20.4	22.3	19.1	20.0	
Poverty	—	19.5	18.5	19.1	16.7	14.9	
Severe poverty	—	9.9	5.1	6.5	6.1	3.7	
BMI (%)							0.001
Underweight (BMI <18.5)	3.2	2.8	3.1	2.6	3.5	2.8	
Overweight (BMI 25 to <30)	29.9	30.2	33.7	34.2	30.8	32.2	
Obesity (BMI >30)	18.6	18.5	20.6	21.5	23.5	25.9	
Current use of reversible contraceptives^5^	–	15.2	12.2	13.8	15.5	15.3	0.18

Weighted estimates.

1 Encuesta Nacional de Micronutrientes.

2 Sistema de Vigilancia Epidemiológica de Salud y Nutrición.

3 Rao–Scott chi-square test (categorical variables) or ANOVA with Taylor series variance (continuous variables) for differences in distribution within at least one survey cycle.

4 The Multidimensional Poverty Index is an international measure capturing acute deprivations in health, education, and living standards [48]. It is a household-level indicator, although differences between estimates for preschool age children (PSC) and women of reproductive age (WRA) exist because not all households with WRA have PSC.

5 Reversible contraceptives included intrauterine device, contraceptive injection or implant, oral contraceptive, vaginal contraceptive method, or hormonal contraceptive patch.

### Iron and vitamin A in food samples

**FIGURE 1:**
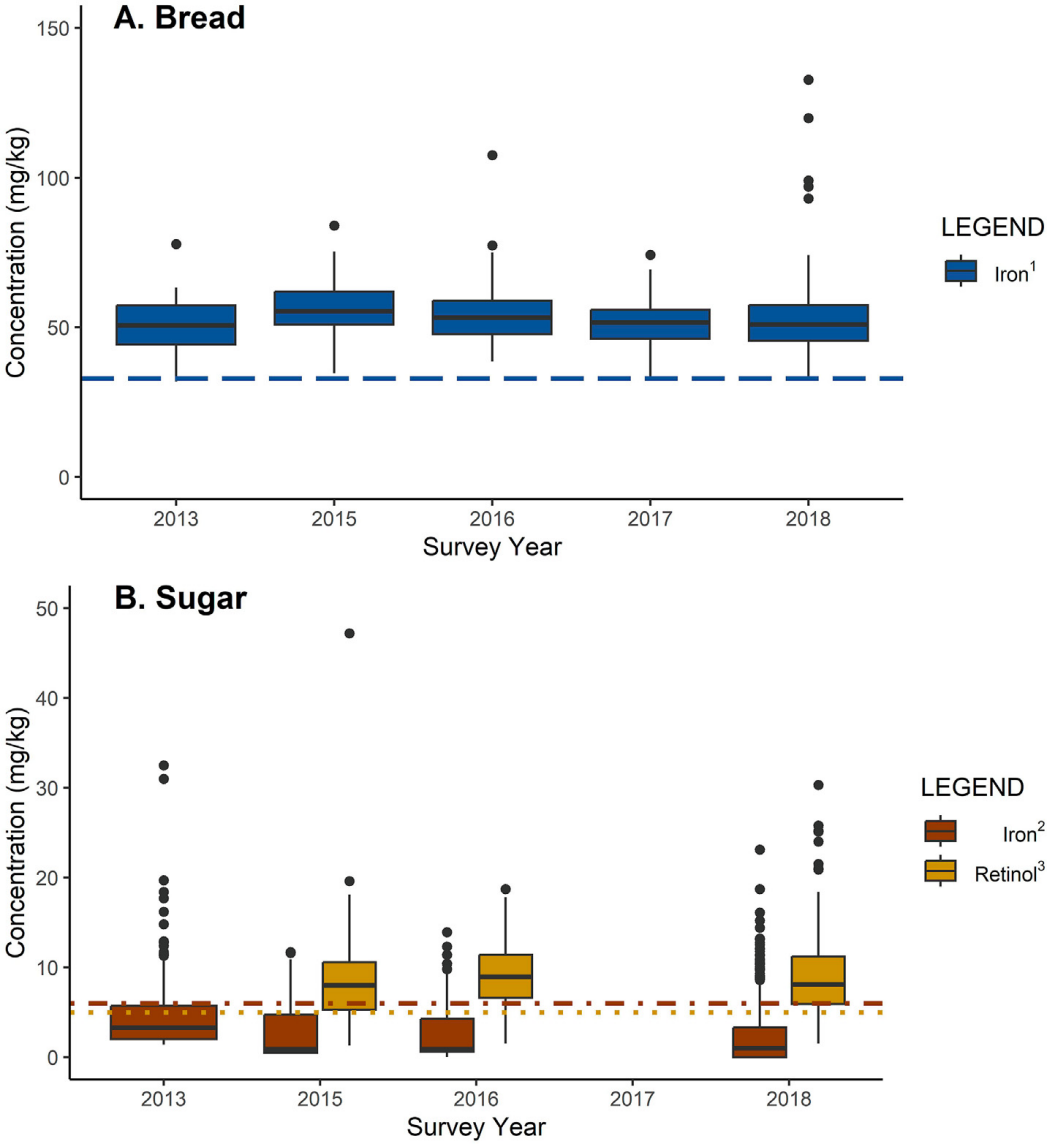
Iron in bread and sugar and retinol in sugar samples from Guatemala, 2013–2018/2019. Unweighted estimates. Kruskal–Wallis test for differences in the distribution of concentration across surveys. ^1^Regulatory minimum: ≥33 mg iron/kg bread assuming 60% wheat flour (2013: *n* = 71, *M* = 50.7 mg/kg; 2015: *n* = 96, *M* = 55.4 mg/kg; 2016: *n* = 96, *M* = 53.3 mg/kg; 2017/2018: *n* = 96, *M* = 51.6 mg/kg; 2018/2019: *n* = 97, *M* = 51.0 mg/kg); *P* < 0.001. ^2^Manufacturer reported minimum: ≥6 mg iron/kg sugar (2013: *n* = 193, *M* = 3.3 mg/kg; 2015: *n* = 207, *M* = 0.9 mg/kg; 2016: *n* = 258, *M* = 0.9 mg/kg; 2018/2019: *n* = 288, *M* = 1.0 mg/kg); *P* < 0.001. ^3^Regulatory minimum: ≥5 mg retinol/kg sugar (2013: N/A; 2015: *n* = 207, *M* = 8.0 mg/kg; 2016: *n* = 258, *M* = 9.0 mg/kg; 2018/2019: *n* = 293, *M* = 8.1 mg/kg); *P* = 0.01.

Figure 1 describes the level of the fortification of bread with iron and of sugar with iron and vitamin A with regulatory and reported minimums shown as reference lines. The median concentration of iron in bread varied significantly across the study period (*P* < 0.001) from a low in 2013 of 50.7 mg/kg to a high in 2015 of 55.4 mg/kg. All bread samples, except 2, exceeded regulatory minimums, suggesting that the wheat flour used to make bread was adequately fortified with iron (data not shown). The median concentration of iron in sugar also varied across the study period (*P* < 0.001) from a high in 2013 of 3.3 mg/kg to a low in 2016 of 0.9 mg/kg. The intrinsic value of iron in standard (white) sugar in Guatemala is between 0.1 and 1 mg/kg [41], and the manufacturer reported the minimum level of iron in fortified sugar is 6 mg/kg [8]. Approximately 20% of sugar samples across all survey years met the manufacturer’s minimum (data not shown), indicating that voluntary iron fortification of sugar occurred in a portion of the samples taken from households. We estimated a median concentration of 5.9 mg iron/kg sugar (IQR: 3.3, 8.4) among samples with >2 mg iron/kg sugar (41.1% of samples), a level double the intrinsic value (data not shown). Retinol concentrations in sugar also varied over the study period (*P* = 0.01) from its lowest median concentration of 8.0 mg/kg in 2015 to highest of 9.0 mg/kg in 2016. Approximately 82% of household sugar samples met the regulatory minimum of 5 mg of retinol/kg of sugar required in retail sugar sales (data not shown) [37].

### Fortifiable foods in households

Supplemental Table 2 describes fortifiable staples found within Guatemalan households. Sugar consumption, especially standard unrefined sugar, is ubiquitous within households. However, there has been a significant decrease in the presence of standard sugar and corresponding increase in the presence of brown sugar within households from 2013 to 2018/2019 (both *P* < 0.001). Among labeled sugar, nearly all were national brands. Sweetened and sandwich bread were present in ∼13% and 10% of households, respectively, across all surveys. Nearly all (>95%) bread was unlabeled. Approximately 2 units of bread were purchased per household per week. Maize flour was present in ∼8% of households, potentially representing how households purchase prepared maize tortillas rather than raw flour. More than 90% of maize flour was labeled, and most were national brands.

### Frequency of consumption of fortifiable foods

Supplemental Figure 1A and B shows the reported weekly consumption of sugars, breads, fortified cereals, and maize flour consumed by PSC and WRA, respectively, in their homes from 2013 to 2018/2019. Sugar consumption was nearly universal among both populations: >95% within each year consumed it 3–7 d/wk. Bread/wheat flour is also commonly consumed, and the distribution of frequency of consumption per week has increased (*P* = 0.008) with 84% of PSC consuming it at least once per week in 2013 and 2015 and 91% consuming it as often in 2017/2018 and 2018/2019. Among WRA, the distribution of reported bread/wheat flour consumption also shifted toward more frequent consumption (*P* < 0.001), with 88% consuming it at least once per week in 2013 and 95% consuming it as often in 2018/2019. The consumption of specialty fortified cereals varied over time among PSC (*P* = 0.01) and among WRA (*P* < 0.001). Fifty-two percent of PSC and 38% of WRA reported consuming specialty fortified cereals at least once per week in 2013, and these proportions increased to 58% and 49%, respectively, in 2018/2019. Reported consumption of maize flour in homemade foods increased significantly only for PSC (*P* = 0.03), although weekly consumption was low for both populations (10%–15%).

### Anemia and micronutrient deficiencies

**FIGURE 2:**
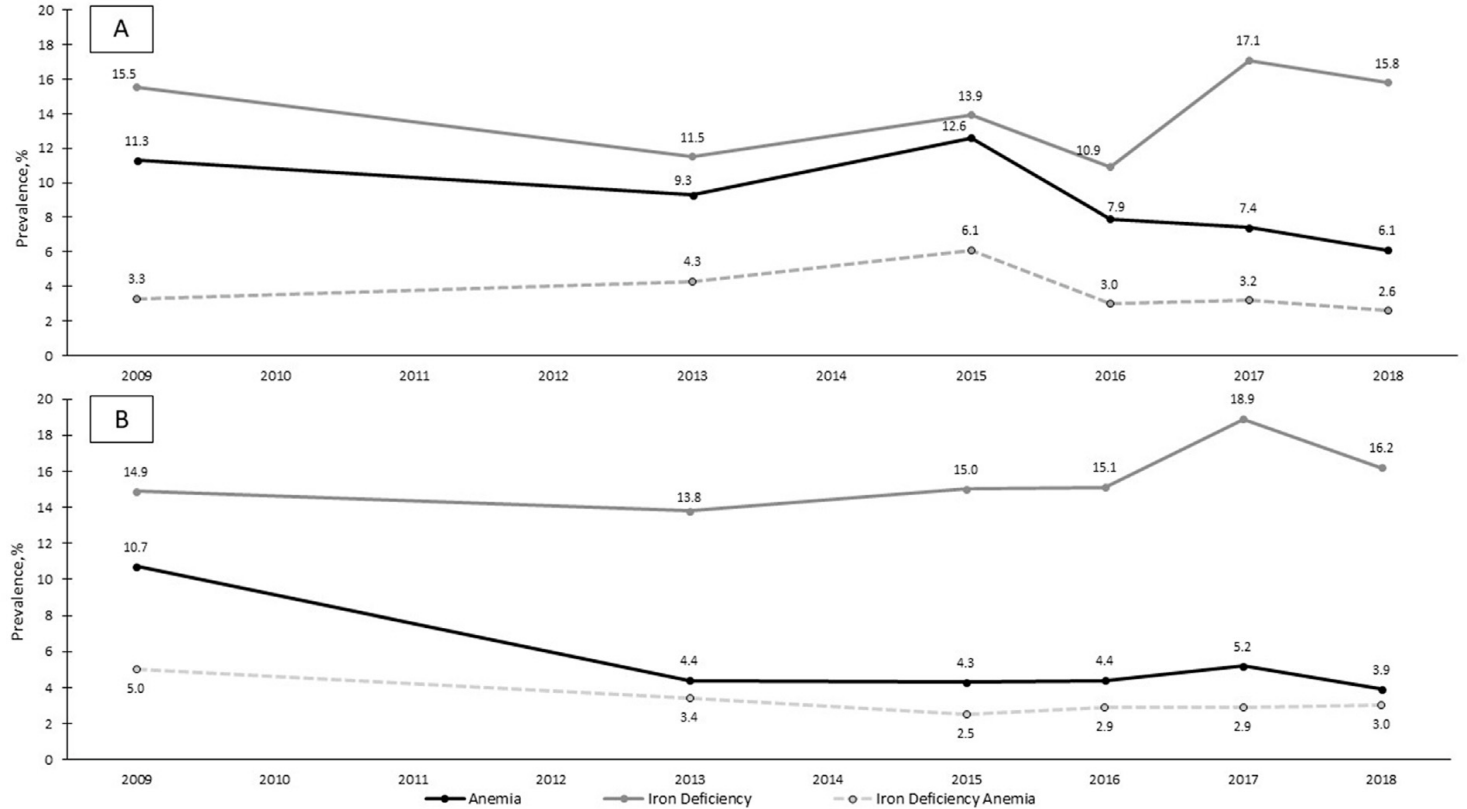
(A) Prevalence of anemia and iron deficiency among preschool age children in Guatemala, 2008/2009–2018/2019. Estimates are weighted; Anemia = hemoglobin concentration (Hb) <11 g/dL (2008/2009: *n* = 742, 2013: *n* = 878, 2015: *n* = 460, 2016: *n* = 452, 2017/2018: *n* = 456, 2018/2019: *n* = 453); −0.4 percentage-point unadjusted change in anemia per year (2008/2009–2018/2019), *P* value for trend = 0.007; iron deficiency = serum ferritin <12 μg/L, adjusted for inflammation (BRINDA) [31] (2008/2009: *n* = 873, 2013: *n* = 859, 2015: *n* = 456, 2016: *n* = 445, 2017/2018: *n* = 470, 2018/2019: *n* = 473); +0.2 percentage-point unadjusted change in iron deficiency per year (2008/2009–2018/2019), *P* value for trend = 0.31; iron deficiency anemia = concurrent anemia and iron deficiency (2008/2009: *n* = 742, 2013: *n* = 858, 2015: *n* = 456, 2016: *n* = 442, 2017/2018: *n* = 446, 2018/2019: *n* = 477), +0.1 percentage-point unadjusted change in iron deficiency anemia per year (2008/2009–2018/2019), *P* value for trend = 0.52. *P* values for trends in micronutrient deficiencies were calculated from generalized linear mixed models with survey year as random intercept. (B) Prevalence of anemia and iron deficiency among nonpregnant women of reproductive age in Guatemala, 2008/2009–2018/2019. Estimates are weighted. Anemia = hemoglobin concentration (Hb) <12 g/dL (2008/2009: *n* = 745, 2013: *n* = 1,672, 2015: *n* = 1123, 2016: *n* = 1197, 2017/2018: *n* = 1201, 2018/2019: *n* = 1268); −0.7 percentage-point unadjusted change in anemia per year (2008/2009–2018/2019), *P* value for trend <0.001; iron deficiency = serum ferritin <15 μg/L, adjusted for inflammation (BRINDA) [31] (2008/2009: *n* = 1323, 2013: *n* = 1640, 2015: *n* = 1131, 2016: *n* = 1194, 2017/2018: *n* = 1258, 2018/2019: *n* = 1266); +0.2 percentage-point unadjusted change in iron deficiency per year (2008/2009–2018/2019), *P* value for trend = 0.08; iron deficiency anemia = concurrent anemia and iron deficiency (2008/2009: *n* = 745, 2013: *n* = 1635, 2015: *n* = 1119, 2016: *n* = 1189, 2017/2018: *n* = 1191, 2018/2019: *n* = 1265); −0.2 percentage-point unadjusted change in iron deficiency anemia per year (2008/2009–2018/2019), *P* value for trend <0.001. *P* values for trends in micronutrient deficiencies were calculated from generalized linear mixed models with survey year as random intercept.

Figure 2A and B shows the prevalence of anemia and iron deficiency among PSC and WRA, respectively. The prevalence of anemia among PSC was 11.3% (8.0, 14.5) in 2008/2009 and 6.1% (3.6, 8.6) in 2018/2019, reflecting a −0.4 percentage-point decline in anemia prevalence per year (*P*-trend = 0.007). The prevalence of anemia among WRA was 10.7% (7.2, 14.2) in 2008/2009 and 3.9% (2.7, 5.2) in 2018/2019, reflecting a −0.7 percentage-point decline in anemia prevalence per year (*P* < 0.001). The prevalence of iron deficiency among PSC was 15.5% (12.1, 19.0) in 2008/2009, 10.9% (7.4, 14.5) in 2016 (lowest estimate), but 17.1% (13.3, 21.0) in 2017/2018 (highest), reflecting no significant 10-y change (*P* = 0.31). The prevalence of iron deficiency among WRA was 14.9% (11.6, 18.2) in 2008/2009, 13.8% (11.8, 15.8) in 2013 (lowest), and 18.9% (16.3, 21.6) in 2017/2018 (highest), reflecting no significant 10-y change (*P* = 0.08). The prevalence of iron deficiency anemia among PSC was 3.3% (1.4, 5.2) in 2008/2009, 6.1% (3.5, 8.6) in 2015 (highest), and 2.6% (13.3, 21.0) in 2018/2019, reflecting no significant 10-y change (*P* = 0.52). The prevalence of iron deficiency anemia among WRA was 5.0% (2.4, 7.7) in 2008/2009 and 3.0% (1.9, 4.1) in 2018/2019, reflecting a −0.2 percentage-point decline per year (*P* < 0.001).

Supplemental Figure 2A and B shows the prevalence of anemia-related micronutrient deficiencies where available at each survey among PSC and WRA, respectively. Among PSC, the prevalence of elevated sTfR, a marker of iron deficient erythropoiesis, was 4.0% (2.4, 5.5) in 2013 and 10.7 (7.9, 13.5) in 2018/2019, whereas the prevalence of elevated sTfR among WRA stayed at ∼2% over the same period. The prevalence of vitamin A deficiency among PSC was 2.3% (0.3, 4.3) in 2015 and lower in all other years examined, and among WRA it was 0.6% (0.0, 1.1) in 2017/2018 and lower in all other years examined. At the only measurement, there was no folate deficiency among PSC or WRA in 2008/2009 (data not shown). Based on limited data from 2008/2009, 2013, and 2016, zinc deficiency was above or just below the cut-point for classification as a problem of public health concern (>20%) in both populations [42]. The prevalence of low vitamin B12 among PSC was 18.9% (13.8, 24.0) in 2008/2009 and 19.3% (15.6, 23.0) in 2016, and among WRA it was 18.2% (15.1, 21.3) in 2008/2009 and 13.2% (11.3, 15.1) in 2016.

Among PSC in Guatemala, there was no unadjusted change in altitude-corrected mean Hb between 2008/2009 and 2018/2019 (*P*-trend = 0.30) and a +0.05 g/dL increase in mean Hb per year during 2013 to 2018/2019 (*P*-trend < 0.001). Table 3 shows potential factors associated with Hb and ferritin among PSC in Guatemala. A higher frequency of reported bread/wheat flour consumption was positively associated with Hb. High-dose vitamin A supplementation during the previous 6 mo had an unexpected negative association with Hb. There was no significant change in mean ferritin from 2008/2009 to 2018/2019 (*P*-trend = 0.59) and a −2.79% unadjusted decrease in mean ferritin per year during 2013 to 2018/2019 (*P*-trend < 0.001). Fortifiable food consumption was not associated with ferritin. The consumption of MNPs in the previous 3 mo had an unexpected negative association with ferritin, though adherence was unknown.

**TABLE 3:** Trends in hemoglobin and serum ferritin concentration among preschool age children in Guatemala, 2013–2018/2019

Fixed effects	Hemoglobin (g/dL) (95% CI)^1^ (*n* = 2641)	Ferritin (%) (95% CI)^2^ (*n* = 2664)
Age, 6–24 vs. 25–59 mo	−0.65 (−0.73, −0.57)∗∗∗	−47.73 (−53.32, −42.15)∗∗∗
Male vs. female	−0.05 (−0.12, 0.02)	−4.56 (−9.49, 0.37)∼
Indigenous vs. not indigenous	0.00 (−0.08, 0.08)	2.94 (−2.45, 8.33)
Urban vs. rural	0.13 (0.04, 0.21)∗∗	11.69 (5.84, 17.54)∗∗∗
Multidimensional Poverty Index		
Vulnerable vs. no poverty	−0.01 (−0.12, 0.09)	0.87 (−6.26, 8.01)
Poverty vs. no poverty	−0.10 (−0.21, 0.00)∗	−7.40 (−14.50, −0.30)∗
Severe vs. no poverty	−0.12 (−0.24, 0.01)∼	−5.27 (−14.00, 3.45)
Receives remittances	−0.04 (−0.15, 0.07)	4.07 (−3.62, 11.76)
BMI-for-age *z*-score	0.03 (0.00, 0.06)∗	−1.53 (−3.51, 0.45)
Length-for-age/height-for-age *z*-score	0.13 (0.09, 0.17)∗∗∗	2.00 (−0.54, 4.54)
Retinol-binding protein (μmol/L)	0.48 (0.38, 0.59)∗∗∗	32.96 (25.51, 40.40)∗∗∗
Frequency of consumption of fortifiable foods during previous 7 d		
Standard sugar: 1–2 vs. 0 d	0.04 (−0.17, 0.24)	−6.62 (−21.12, 7.88)
Standard sugar: 3–7 vs. 0 d	0.08 (−0.05, 0.22)	−1.29 (−10.49, 7.91)
Bread or wheat flour: 1–2 vs. 0 d	0.13 (0.00, 0.25)∗	−1.70 (−10.33, 6.93)
Bread or wheat flour: 3–7 vs. 0 d	0.26 (0.14, 0.37)∗∗∗	5.28 (−2.95, 13.50)
Maize flour: 1–2 vs. 0 d	−0.12 (−0.27, 0.04)	1.92 (−9.00, 12.84)
Maize flour: 3–7 vs. 0 d	−0.01 (−0.19, 0.17)	5.90 (−6.53, 18.32)
Fortified cereals: 1–2 vs. 0 d	0.09 (−0.01, 0.18)∼	0.60 (−6.11, 7.31)
Fortified cereals: 3–7 vs. 0 d	−0.01 (−0.09, 0.07)	4.07 (−1.68, 9.81)
High-dose vitamin A supplement, previous 6 mo	−0.10 (−0.20, −0.01)∗	
Multiple micronutrient powders, previous 3 mo	0.02 (−0.06, 0.11)	−6.78 (−12.40, −1.16)∗
Iron supplement, previous 3 mo	−0.01 (−0.18, 0.16)	−5.74 (−16.46, 4.98)
Folic acid supplement, previous 3 mo	−0.01 (−0.19, 0.18)	
Random effects		
2013	0.13 (0.07, 0.20)	9.28 (4.48, 14.19)∗
2015	−0.13 (−0.20, −0.07)	−0.13 (−6.08, 6.16)
2016	−0.11 (−0.20, −0.02)	5.00 (−0.03, 10.33)
2017/2018	−0.04 (−0.14, 0.05)	−11.79 (−17.92, −6.12)∗
2018/2019	0.15 (0.08, 0.22)	−2.36 (−8.23, 3.47)

^3^∼*P* < 0.10; ∗*P* < 0.05; ∗∗*P* < 0.01; ∗∗∗*P* < 0.001.

^4^95% CI are based on bootstrapped estimates for random effects.

1 Generalized linear mixed model of the association of fortifiable food consumption and micronutrient supplementation with altitude-corrected hemoglobin adjusted for age, sex, ethnicity, rurality, BMI-for-age *z*-score, height-for-age *z*-score, poverty, remittances, retinol-binding protein, and form of blood draw (capillary or venous). +0.01 g/dL unadjusted change in mean Hb per year (2008/2009–2018/2019), *P* value for trend = 0.30; +0.05 g/dL unadjusted change in mean Hb per year (2013-2018/2019), *P* value for trend <0.001.

2 Generalized linear mixed model of the association of fortifiable food consumption and micronutrient supplementation with serum ferritin corrected for inflammation (BRINDA) and log-transformed adjusted for age, sex, ethnicity, rurality, BMI-for-age *z*-score, height-for-age *z*-score, poverty, remittances, and retinol-binding protein adjusted for inflammation. 0.17% unadjusted change in mean ferritin per year (2008/2009–2018/2019), *P* value for trend = 0.59; −2.79% unadjusted change in mean ferritin per year (2013–2018/2019), *P* value for trend <0.001.

Among WRA in Guatemala, there was a −0.02 g/dL unadjusted decrease in altitude-corrected mean Hb per year during 2008/2009 to 2018/2019 (*P*-trend < 0.001) and a −0.04 g/dL decrease in mean Hb per year during 2013 to 2018/2019 (*P*-trend < 0.001). Table 4 shows potential factors associated with Hb and ferritin among WRA in Guatemala. Consuming standard sugar 3–7 d compared with 0 d/wk was positively associated with Hb. There was a −1.38% unadjusted decrease in mean ferritin concentration from 2008/2009 to 2018/2019 (*P*-trend < 0.001) and a −1.64% decrease in mean ferritin during 2013 to 2018/2019 (*P*-trend = 0.002). Fortifiable food consumption was not associated with ferritin. There was a positive association between current use of reversible contraceptives, such that WRA users had on average and over the years had, higher levels of hemoglobin (+0.21 g/dL) and ferritin (+18.9 μg/L) relative to noncurrent users (each adjusted *P* < 0.001).

**TABLE 4:** Trends in hemoglobin and serum ferritin concentration among nonpregnant women of reproductive age in Guatemala, 2013–2018/2019

Fixed effects	Hemoglobin (g/dL) (95% CI)^1^ (*n* = 5957)	Ferritin (%) (95% CI)^2^ (*n* = 6031)
Age (y)	0.00 (0.00, 0.00)	0.40 (0.11, 0.69)∗∗
Parity	−0.04 (−0.06, −0.02)∗∗∗	−1.47 (−2.73, −0.21)∗
Indigenous vs. not indigenous	0.01 (−0.05, 0.08)	−1.42 (−5.78, 2.93)
Urban vs. rural	0.02 (−0.04, 0.09)	0.98 (−3.41, 5.37)
Multidimensional Poverty Index		
Vulnerable vs. no poverty	−0.08 (−0.15, 0.00)∼	−0.95 (−6.17, 4.27)
Poverty vs. no poverty	−0.09 (−0.18, −0.01)∗	−11.11 (−16.86, −5.36)∗∗∗
Severe vs. no poverty	−0.13 (−0.26, 0.00)∼	−12.93(−21.71, −4.14)∗∗
Receives remittances	−0.05 (−0.14, 0.05)	−6.61 (−12.70, −0.52)∗
Years of schooling	0.00 (−0.01, 0.01)	−0.32 (−0.89, 0.25)
BMI		
Underweight (BMI <18.5 kg/m^2^) vs. normal weight	0.04 (−0.14, 0.21)	12.68 (0.93, 24.43)∗
Overweight (BMI 25 to <30 kg/m^2^) vs. normal weight	0.12 (0.05, 0.19)∗∗∗	6.18 (1.42, 10.94)∗
Obesity (BMI >30 kg/m^2^) vs. normal weight	0.21 (0.12, 0.29)∗∗∗	21.18 (15.67, 26.69)∗∗∗
Retinol-binding protein (μmol/L)	0.19 (0.14, 0.23)∗∗∗	23.71 (20.58, 26.85)∗∗∗
Current use of reversible contraceptives	0.21 (0.13, 0.29)∗∗∗	18.91 (13.39, 24.44)∗∗∗
Frequency of consumption of fortifiable foods during previous 7 d		
Standard sugar: 1–2 vs. 0 d	0.11 (−0.07, 0.28)	−7.77 (−19.61, 4.07)
Standard sugar: 3–7 vs. 0 d	0.13 (0.03, 0.23)∗	−4.66 (−11.34, 2.03)
Bread or wheat flour: 1–2 vs. 0 d	−0.02 (−0.13, 0.09)	2.59 (−5.04, 10.21)
Bread or wheat flour: 3–7 vs. 0 d	−0.05 (−0.16, 0.05)	−1.43 (−8.62, 5.77)
Maize flour: 1–2 vs. 0 d	−0.09 (−0.20, 0.02)	0.36 (−7.18, 7.90)
Maize flour: 3–7 vs. 0 d	−0.11 (−0.24, 0.01)∼	2.65 (−5.66, 10.96)
Fortified cereals: 1–2 vs. 0 d	0.00 (−0.08, 0.07)	−4.33 (−9.18, 0.53)∼
Fortified cereals: 3–7 vs. 0 d	−0.08 (−0.15, 0.00)∼	−4.13 (−9.36, 1.10)
Random effects		
2013	0.02 (−0.01, 0.08)	8.61 (4.27, 12.97)∗
2015	−0.02 (−0.08, 0.01)	2.32 (−1.02, 6.50)
2016	−0.03 (−0.08, 0.01)	−2.31 (−6.14, 1.11)
2017/2018	−0.02 (−0.07, 0.01)	−8.01 (−12.56, −3.65)∗
2018/2019	0.05 (0.00, 0.11)	−0.62 (−5.09, 3.82)

^3^ ∼*P* < 0.10; ∗*P* < 0.05; ∗∗*P* < 0.01; ∗∗∗*P* < 0.001.

^4^95% CI are based on bootstrapped estimates for random effects.

1 Generalized linear mixed model of the association of fortifiable food consumption with altitude- and smoking-corrected hemoglobin adjusted for age, parity, ethnicity, rurality, poverty, remittances, education, BMI, retinol-binding protein, and reversible contraceptives. −0.02 g/dL unadjusted change in mean Hb per year (2008/2009–2018/2019), *P* value for trend <0.001; −0.04 g/dL unadjusted change in mean Hb per year (2013–2018/2019), *P* value for trend <0.001.

2 Generalized linear mixed model of the association of fortifiable food consumption and micronutrient supplementation with serum ferritin corrected for inflammation (BRINDA) and log-transformed adjusted for age, parity, ethnicity, rurality, poverty, remittances, education, BMI, retinol-binding protein, and reversible contraceptives. 0.17% unadjusted change in mean ferritin per year (2008/2009–2018/2019), *P* value for trend = 0.59; −1.38% change in mean ferritin per year (2008/2009–2018/2019), *P* value for trend <0.001; −1.64% change in mean ferritin per year (2013–2018/2019), *P* value for trend = 0.002.

In adjusted fixed effects models of Hb and ferritin for PSC and WRA, there was no significant interaction between reported consumption of any fortifiable food and survey year indicating that the associations did not differ by survey year (data not shown).

## Discussion

Guatemala has implemented multiple food fortification strategies with mandates on enrichment of wheat and nixtamalized maize flours with iron and other anemia-related micronutrients, and sugar fortification with vitamin A. Among PSC, Guatemala implements several supplementation programs, including iron, folic acid, and multiple micronutrient supplements, whereas in recent years, high-dose vitamin A supplementation has been limited to children aged 6–11 mo. The institutionalization of fortification and supplementation policies has coincided with declines in the prevalence of anemia among PSC and WRA over many years and the prevalence of iron deficiency anemia declined slightly among WRA. However, iron status did not improve during the 10-y period between 2008/2009 and 2018/2019 and worsened in 2017/2018. Fortification and micronutrient supplementation were not associated with improved ferritin status, and we were unable to identify clear time-dependent and individual-level correlates of the recent increases in iron deficiency among PSC and WRA. The observed negative association between consumption of MNPs and ferritin was unexpected, and possibly due to confounding by indication of treatment [43] or enhanced MNP program implementation in areas of higher risks. However, the intensity of micronutrient supplementation interventions overall declined precipitously during the study period. Furthermore, young children aged 6 mo to 5 y are generally not expected to consume sufficient quantities of bread to be the primary beneficiary of the industrial fortification of wheat flour or maize flour, and previous studies have shown that wheat flour is not frequently consumed by the poorest, rural, and indigenous populations [44]. Micronutrient supplementation and food fortification likely have more difficulty reaching rural and indigenous populations, which consume less industrially fortified flours and are often at a higher risk of iron deficiency [44].

The frequency of wheat flour consumption was positively associated with a higher Hb among PSC over the 2013–2018/2019 period in which there was a significant increasing trend in Hb concentrations. In contrast, there was a significant decreasing trend in Hb among WRA during the same period. Among WRA, reported standard sugar consumption was positively associated with Hb, potentially reflecting the role of vitamin A fortification in anemia prevention [9,45]. The plausibility of this pathway is strengthened by the strong association of RBP, a marker of vitamin A status, with Hb. Expected improvements in micronutrient status due to the introduction of maize flour fortification in 2016 may not have been observable during the survey because of typical delays in program maturation.

The iron fortification of bread met regulatory minimums for wheat flour fortification, and iron was present in fortification levels in one-fifth of sugar samples collected. Vitamin A fortification levels were also acceptable in over 80% of sugar samples. Current use of reversible contraceptives was positively associated with Hb and ferritin, and aligned with previous studies finding use of hormonal contraceptives as protective against anemia [46]. These positive indicators of fortification programs and few explanatory individual-level indicators suggest that there are other ecologic explanations for the decline in Hb and ferritin values beyond those measured by this study. Although the surveillance system observed a decline in poverty in Guatemala, other national metrics show an increase in poverty during the first portion of the study period (2006–2014) [47]. Larger economic and social factors may have contributed to the observed population-level changes in Hb and ferritin concentrations.

This study is strengthened by its data source, 6 nationally representative surveys over 10 y. These data sources are rich in variables pertaining to food fortification, supplementation, and micronutrient status, and used many comparable methods across time including the same laboratory, methodology, and many of the same well-trained personnel. These findings are likely generalizable to most of the 900,000 PSC and 4 million WRA in Guatemala. The variety of micronutrient indicators allows a more complete examination of the factors associated with anemia in the studied populations. This study also benefits from the use of food sample data to examine the level of fortification in key fortifiable foods available to the target populations. The use of the MPI is another strength as it captures many differing dimensions of poverty and aligns with Sustainable Development Goals and indicators [48]. Our analysis results are also consistent with external MPI estimates of the same period indicating that the SIVESNU nationally representative surveys adequately captured the demography of the country [15].

This study has several limitations. The ENMICRON survey, which occurred before the SIVESNU surveillance system was established, did not collect information on consumption of fortifiable foods, which meant this survey was dropped from relevant models. There were differences in the assessment of the biomarkers of nutritional status and inflammation between the 2008/2009 ENMICRON survey and the SIVESNU surveillance system, limiting their comparability. The blood source varied in 2013 and 2015 SIVESNU cycles (pooled capillary blood) compared with other years (venous blood). Food security could not be included in analyses due to the inconsistent use of tools across surveys. Food samples data could not be incorporated into the model because data were available for only a small subsample of households. The fortifiable food consumption frequency was evaluated over the previous 7 d and may not reflect usual consumption, though the consumption of fortifiable staple foods is less seasonally dependent, and the surveys were usually collected over 6–8 mo crossing multiple seasons. The food frequency data are also limited to foods prepared in the household and precludes potential interpretations related to street foods and those eaten outside. This is especially limiting to maize flour, which is most often consumed as premade purchased tortillas. Although the mandate for the fortification of nixtamalized maize flour was implemented during the study period, no maize flour samples were examined for micronutrient content. The surveys did not have dietary recall data or asked about the amounts of fortified food consumed, which is another limitation.

Guatemala has implemented multiple food fortification and micronutrient supplementation strategies to prevent several micronutrient deficiencies, including iron. The intensity of micronutrient supplementation interventions among PSC has declined between 2013 and 2018/2019. Although the country has made progress in the reduction of anemia among PSC, recent decreasing trends in ferritin among PSC and WRA, and Hb among WRA are concerning and may warrant renewed intervention in populations with higher risk and further monitoring. Secular trends toward poverty alleviation, education, and developmental might be responsible for changes not explained by the micronutrient interventions evaluated. Examining trends over time in both micronutrient program implementation quality and fidelity, in conjunction with micronutrient status assessment is useful for understanding program effectiveness, sustainability, safety, and accountability. These data are important for making evidence-based decisions to adapt, scale-up, or scale-down public health micronutrient delivering programs to improve their effectiveness and efficiency.

## Author contributions

The authors’ responsibilities were as follows – MP, KM: collected date and oversaw all technical aspects and training and were involved in the budgeting and acquisition of supplies/materials for the system. MP, DIM, CM, MEDJ, and RF-A: involved in the original design of the surveillance system. DIM, CM: provided technical assistance. MEDJ, RF-A, OYA, LG: provided in-kind technical assistance. MCS, LG, OYA: were advisors to the surveillance system and all authors: read and approved the final manuscript.

## Conflict of Interest

MP and KM were employed by the SIVESNU surveillance system for the data collection cycles reported in this manuscript and oversaw all technical aspects and training and were involved in the budgeting and acquisition of supplies/materials for the system. The other authors declare no other conflicts of interest.

## Funding

The SIVESNU surveillance system described in this manuscript received funding and/or in-kind office space, laboratory installations, or technical assistance from the INCAP, USAID, and CDC (2013, 2015, 2016, 2017/2018, and 2018/2019 cycles), UNICEF (2016, 2017/2018, 2018/2019 cycles), and World Food Programme (2013, 2015 cycles). MP and KM were employees of the surveillance system during the 2013–2018/2019 cycles. We declare no external funding for the preparation of this manuscript. Additional authors work for agencies that provided funding, in-kind technical assistance, advice, or Government of Guatemala leadership for the system.

The findings and conclusions in this manuscript are those of the authors and do not necessarily represent the official position of the CDC, INCAP, USAID, or UNICEF.

## Data availability

Datasets described in the manuscript and SIVESNU reports for 2013, 2015, 2016, 2017/2018, and 2018/2019 cycles are available at https://www.siinsan.gob.gt/siinsan/monitoreo-y-evaluacion/#.

## Declaration of interests

The authors declare the following financial interests/personal relationships which may be considered as potential competing interests: The SIVESNU surveillance system described in this manuscript received funding and/or in-kind office space, laboratory installations, or technical assistance from the INCAP, USAID and CDC (2013, 2015, 2016, 2017/18 2018/19 cycles), UNICEF (2016, 2017/18, 2018/19 cycles), and World Food Programme (2013, 2015 cycles). Mireya Palmieri and Karla Mesarina were employees of the surveillance system during the 2013-2018/2019 cycles. All authors work for agencies that provided funding or in-kind technical assistance and advising to the system or providing advising from the Government of Guatemala.
