# Supplementary material for: Trends in Micronutrient Interventions, Anemia, and Iron Deficiency among Women and Children in Guatemala, 2009–2019
Source: Curr Dev Nutr. 2023 Jul 20;7(8):101970. doi: 10.1016/j.cdnut.2023.101970 (PMC10448407; doi:10.1016/j.cdnut.2023.101970)
Supplement: Multimedia component 1 [file mmc1.docx]

| **Supplementary Table 1: Timeline of Food Fortification Laws and Regulations in Guatemala** | | | | |
| --- | --- | --- | --- | --- |
| Year | Food | Nutrient | Fortificant | Minimum Level of Micronutrient (mg/kg) |
| 1954  Revised: 2004 | Salt | Fluoride^1^  Iodine | Potassium or sodium fluoride  Potassium iodate or iodide | 175.0  20.0 |
| 1975  Revised: 2000 | Sugar^2^ | Vitamin A | Retinol | 5.0 |
| 1985^3^  Revised: 2007 | Wheat flour | Folate  Iron  Niacin  Riboflavin  Thiamin | Folic acid  Ferrous fumarate^4^  Niacinamide  Riboflavin  Thiamin mononitrate | 1.8  55.0  55.0  4.2  6.2 |
| 2016  Revised: 2017 | Nixtamalized Maize flour | Vitamin B12  Folate  Iron  Niacin  Riboflavin  Thiamin  Zinc | Cyanocobalamin  Folic acid  Iron bisglycinate  Niacinamide  Riboflavin  Thiamin mononitrate  Zinc bisglycinate | 0.017  0.6  26.2  23.0  1.8  2.2  22.4 |
| Ministry of Public Health and Social Assistance, Government of Guatemala  ^1^ Fluoride mandated in 2004.  ^2^ A leading brand of sugar sold in Guatemala has voluntarily fortified its sugar with iron at a level between 6 and 12 mg/kg since October 2008. As of 2012, this brand accounted for 31.4% of all sugar consumed in the country.  ^3^ Voluntary until 2002.  ^4^ Ferrous fumarate replaced reduced iron in 2001. | | | | |

| Supplementary Table 2: Fortifiable Foods in Guatemalan Households, 2013-2018/19 | | | | | |
| --- | --- | --- | --- | --- | --- |
|  | 2013  *(n*=2,404) | 2015  *(n*=2,301) | 2016  *(n*=2,376) | 2017/18  *(n*=2,424) | 2018/19  *(n*=2,490) |
| Standard sugar |  |  |  |  |  |
| Present in household, % ^1^ *** | 87.6 | 82.3 | 81.0 | 78.6 | 79.2 |
| Quantity purchased, kg/week | 1.1 (0.2, 2.3) | 1.4 (0.2, 3.2) | 1.4 (0.3, 3.4) | 1.1 (0.2, 2.8) | 0.9 (0.2, 2.7) |
| Quantity purchased per capita, g/day | 33.9 (8.1, 67.5) | 40.5 (8.6, 108.0) | 46.3 (9.3, 115.7) | 38.9 (8.1, 92.6) | 32.4 (6.2, 81.0) |
| Labeled – national brand, % | 49.7 | 53.0 | 47.7 | 49.1 | 44.7 |
| Labeled – imported brand, % ^2^ | 0.5 | 0.5 | 0.3 | 0.1 | 0.2 |
| Brown sugar |  |  |  |  |  |
| Present in household, % ^1^ *** | 5.5 | 8.2 | 10.8 | 13.8 | 15.1 |
| Quantity purchased, kg/week | 0.0 (0.0, 0.0) | 0.0 (0.0, 0.0) | 0.0 (0.0, 0.0) | 0.0 (0.0, 0.0) | 0.0 (0.0, 0.2) |
| Quantity purchased per capita, g/day | 0.0 (0.0, 0.0) | 0.0 (0.0, 0.0) | 0.0 (0.0, 0.0) | 0.0 (0.0, 0.0) | 0.0 (0.0, 6.7) |
| Labeled – national brand, % | 42.0 | 37.1 | 32.0 | 31.5 | 25.9 |
| Labeled – imported brand, % ^2^ | 0.8 | 1.2 | 1.8 | 0.0 | 0.0 |
| Refined sugar |  |  |  |  |  |
| Present in household, % | 0.4 | 0.3 | 0.3 | 0.2 | 0.2 |
| Quantity purchased, kg/week | 0.0 (0.0, 0.0) | 0.0 (0.0, 0.0) | 0.0 (0.0, 0.0) | 0.0 (0.0, 0.0) | 0.0 (0.0, 0.0) |
| Quantity purchased per capita, g/day | 0.0 (0.0, 0.0) | 0.0 (0.0, 0.0) | 0.0 (0.0, 0.0) | 0.0 (0.0, 0.0) | 0.0 (0.0, 0.0) |
| Labeled – national brand, % | 22.2 | 33.3 | 42.9 | 33.3 | 60.0 |
| Labeled – imported brand, % ^2^ | 0.0 | 0.0 | 0.0 | 0.0 | 0.0 |
| Sweetened bread |  |  |  |  |  |
| Present in household, % | 12.8 | 12.3 | 12.8 | 13.0 | 13.8 |
| Quantity purchased, units/week | 2.6 (1.1, 6.0) | 2.6 (1.1, 6.0) | 2.9 (1.1, 6.4) | 2.3 (1.1, 5.7) | 2.3 (0.9, 5.1) |
| Quantity purchased per capita, units/day | 0.1 (0.0, 0.2) | 0.1 (0.0, 0.2) | 0.1 (0.0, 0.2) | 0.1 (0.0, 0.2) | 0.1 (0.0, 0.2) |
| Labeled – national brand, % | 1.0 | 1.2 | 0.0 | 1.5 | 1.0 |
| Labeled – imported brand, % ^2^ | 0.0 | 0.0 | 0.0 | 0.0 | 0.0 |
| Sandwich bread |  |  |  |  |  |
| Present in household, % | 9.9 | 10.7 | 8.7 | 9.9 | 10.7 |
| Quantity purchased, units/week | 2.0 (0.7, 4.6) | 2.0 (0.7, 5.0) | 2.3 (0.9, 6.0) | 2.0 (0.9, 5.0) | 2.1 (0.7, 5.0) |
| Quantity purchased per capita, units/day | 0.1 (0.0, 0.1) | 0.1 (0.0, 0.2) | 0.1 (0.0, 0.2) | 0.1 (0.0, 0.2) | 0.1 (0.0, 0.2) |
| Labeled – national brand, % | 3.1 | 4.7 | 3.5 | 2.5 | 2.2 |
| Labeled – imported brand, % ^2^ | 0.9 | 0.0 | 0.0 | 0.0 | 0.0 |
| Maize flour |  |  |  |  |  |
| Present in household, % ^1^ *** | 8.0 | 10.2 | 7.8 | 6.4 | 8.2 |
| Quantity purchased, kg/week | 0.0 (0.0, 0.0) | 0.0 (0.0, 0.1) | 0.0 (0.0, 0.1) | 0.0 (0.0, 0.1) | 0.0 (0.0, 0.1) |
| Quantity purchased per capita, g/day | 0.0 (0.0, 1.3) | 0.0 (0.0, 2.8) | 0.0 (0.0, 2.7) | 0.0 (0.0, 2.3) | 0.0 (0.0, 4.6) |
| Labeled – national brand, % | 67.9 | 83.5 | 72.4 | 61.3 | 72.7 |
| Labeled – imported brand, % ^2^ | 24.1 | 14.3 | 21.8 | 32.4 | 19.7 |
| Unweighted estimates. Estimates are % or median (IQR).  ^1^ Chi-squared test for differences across surveys. ***p<.001  ^2^ Observed; unlabeled foods account for the remainder. | | | | | |

**Supplementary Figure 1A: Frequency of Consumption of Fortifiable Foods in Homemade Meals Among Children 6-59 Months of Age in Guatemala**

Note: Weighted estimates; Rao-Scott chi-square test for differences in consumption of foods across survey years; **p<.01; *p<.05; Fortified cereals include VitaCereal and Incaparina®

**Supplementary Figure 1B: Frequency of Consumption of Fortifiable Foods in Homemade Meals Among Non-Pregnant Women 15-49 Years of Age in Guatemala**

Note: Unweighted estimates; Rao-Scott chi-square test for differences in consumption of foods across survey years; ***p<.001; **p<.01; Fortified cereals include VitaCereal and Incaparina®

**Supplementary Figure 2A: Prevalence of Anemia and Micronutrient Deficiencies among Pre-school Age Children in Guatemala, 2008/09-2018/19**

Note: Weighted estimates; Anemia= hemoglobin concentration (Hb) <11 g/dL (2008/09: *n*=742, 2013: *n*=878, 2015: n=460, 2016: *n*=452, 2017/18: *n*=456, 2018/19: *n*=453); Iron Deficiency= serum ferritin <12 µg/L, adjusted for inflammation (BRINDA)(31) (2008/09: *n*=873, 2013: *n*=859, 2015: *n*=456, 2016: *n*=445, 2017/18: *n*=470, 2018/19: *n*=473); Iron deficiency anemia= concurrent anemia and iron deficiency (2008/09: *n*=742, 2013: *n*=858, 2015: *n*=456, 2016: *n*=442, 2017/18: *n*=446, 2018/19: *n*=477); Low Vitamin B12= serum B12 <200 pg/mL (2008/09: *n*=826, 2016: *n*=525); Vitamin A Deficiency= serum retinol <0.7µmol/L (2008/09) or retinol binding protein <0.7µmol/L as retinol equivalents (2013-2018), adjusted for inflammation (BRINDA)(34) (2008/09: *n*=858, 2013: *n*=859, 2015: *n*=456, 2016: *n*=445, 2017/18: *n*=470, 2018/19: *n*=473); Zinc Deficiency= serum zinc <65 µg/dL, non-fasting samples, adjusted for inflammation (BRINDA)(35)(2008/09: *n*=843, 2013: *n*=65, 2016: *n*=80); Elevated soluble transferrin receptor (sTfR) >8.3 mg/L adjusted for inflammation (BRINDA)(33) (2013: *n*=859, 2015: *n*=456, 2016: *n*=445, 2017/18: *n*=470, 2018/19: *n*=473). Folate deficiency= serum folate <3 ng/mL, which was not present in any children when measured in 2008/09 (*n*=875).

**Supplementary Figure 2B: Prevalence of Anemia and Micronutrient Deficiencies among Non-Pregnant Women of Reproductive Age in Guatemala, 2008/09-2018/19**

Note: Weighted estimates; Anemia= hemoglobin concentration (Hb) <12 g/dL (2008/09: *n*=745, 2013: *n*=1,672, 2015: n=1,123, 2016: *n*=1,197, 2017/18: *n*=1,201, 2018/19: *n*=1,268); Iron Deficiency= serum ferritin <15 µg/L, adjusted for inflammation (BRINDA)(31) (2008/09: *n*=1,323, 2013: *n*=1,640, 2015: *n*=1,131, 2016: *n*=1,194, 2017/18: *n*=1,258, 2018/19: *n*=1,266); Iron deficiency anemia= concurrent anemia and iron deficiency (2008/09: *n*=745, 2013: *n*=1,635, 2015: *n*=1,119, 2016: *n*=1,189, 2017/18: *n*=1,191, 2018/19: *n*=1,265); Low Vitamin B12= serum B12 <200 pg/mL (2008/09: *n*=1,314, 2016: *n*=1,485); Vitamin A Deficiency= serum retinol binding protein <0.7µmol/L as retinol equivalents (2013: *n*=1,640, 2015: *n*=1,132, 2016: *n*=1,197, 2017/18: *n*=1,266, 2018/19: *n*=1,264); Zinc Deficiency= serum zinc <66 µg/dL, non-fasting samples (2013: *n*=82, 2016: *n*=126); Elevated soluble transferrin receptor (sTfR) >8.3 mg/L adjusted for inflammation (BRINDA)(33) (2013: *n*=1,737, 2015: *n*=1,132, 2016: *n*=1,197, 2017/18: *n*=1,266, 2018/19: *n*=1,264);. Folate deficiency= plasma folate <3 ng/mL, was not present when measured in 2008/09 (*n*=1,419).
